# Supplementary material for: Association of single nucleotide polymorphisms in the NRF2 promoter with vascular stiffness with aging
Source: PLoS One. 2020 Aug 11;15(8):e0236834. doi: 10.1371/journal.pone.0236834 (PMC7418968; doi:10.1371/journal.pone.0236834)
Supplement: S1 File — (PDF) [file pone.0236834.s001.pdf]

S1 File

The questionnaires of self-reported sex, age, clinical history, medication history of hypertensive medicine and smoking history in Iwaki Health Promotion Project

*The top row is in the original language (Japanese) and the bottom row is in the translation language (English).

Original language version (Japanese)

基礎情報/Basic information

・性別　　　　　　　□男　　　　□女

・生年月日　　　　　大正・昭和・平成（　　　　）年（　　　）月（　　　　）日

既往歴・服薬歴/Clinical history

・現在治療中の病気、または今までに次の質問の病気で医師にかかったことがありますか。

□ はい

□ いいえ

・現在医師にかかっている方は「治療中」欄の□に、これまでに医師にかかったことのある方は「かかったことがある」欄の□に☑をつけて下さい。

病名	治療中	かかったことがある
脳の病気（　　　　　　　　　　）	□	□
心臓の病気（　　　　　　　　　　）	□	□
胃腸の病気（　　　　　　　　　　）	□	□
肝臓の病気（　　　　　　　　　　）	□	□
腎臓の病気（　　　　　　　　　　）	□	□
骨・関節の病気（　　　　　　　　　　）	□	□
・骨粗鬆症	□	□
・骨折：　□なし		
□あり ⇒ 部位・時期（例：右手首・10 歳　　　　　　　　　　）		
耳の病気（　　　　　　　　　　）	□	□
鼻の病気（慢性副鼻腔炎（蓄膿症)、アレルギー性鼻炎（花粉症を含む）など　　　　）	□	□
アトピー性皮膚炎	□	□
気管支喘息	□	□
糖尿病	□	□
高血圧	□	□
脂質異常症（コレステロール、中性脂肪）	□	□
尿路結石（腎結石、尿管結石）	□	□
認知症	□	□
関節リウマチ	□	□
その他の病気		
①	□	□
②	□	□
③	□	□

・現在、医師に処方されて服用している薬はありますか。 (市販薬・サプリメントは含まない)

☐ はい ⇒ 「はい」の方は、何の薬を服用しているか、☐の中に☑を記入してください。

☐ いいえ

☐高血圧治療薬　☐高脂血症治療薬 (コレステロールの薬)　☐糖尿病治療薬

☐睡眠薬　☐鎮痛剤・解熱剤　☐アレルギー治療薬　☐狭心症治療薬

☐下剤 (便秘の薬)　☐骨粗鬆症治療薬　☐リウマチ治療薬　☐副腎皮質ステロイド

☐抗生剤　☐胃潰瘍治療薬　☐認知症治療薬

☐かぜ薬 (薬剤名　　　　　　　　　　　　　　　　　　　　　　　)

☐漢方薬 (薬剤名　　　　　　　　　　　　　　　　　　　　　　　)

☐その他 (薬剤名　　　　　　　　　　　　　　　　　　　　　　　)

喫煙歴/Smoking history

・現在の喫煙状況について答えてください。

☐吸わない

☐吸っている

☐以前は吸っていた

Translation language version (English)

Basic information

・Sex　☐Male　☐Female

・Date of birth　Day␣␣ / Month␣␣ / Year␣␣␣␣

Clinical history

・Do you have any history of the next question?

☐ Yes

☐ No

・If you are currently undergoing treatment, please check the "Currently under treatment" ☐, and if you have had treatment in the past, please check the "Past treatment" ☐.

Disease/Illness	Current under treatment	Past treatment
Brain disease (　　　　)	☐	☐
Heart disease (　　　　)	☐	☐
Gastrointestinal illness (　　　　)	☐	☐
Liver disease (　　　　)	☐	☐
Kidney disease (　　　　)	☐	☐
Bone and joint disease (　　　　)	☐	☐
・Osteoporosis	☐	☐
・Fracture：☐ No		
☐Yes ⇒ Cite/Period (ex: right wrist/10 years old　　　　)		

Ear disease ( )	☐	☐
Nasal disease [chronic sinustis (maxillary empyema), allergic rhinitis (including hay fever), etc.]	☐	☐
Atopic dematitis	☐	☐
Bronchial asthma	☐	☐
Diabetes	☐	☐
Hypertension	☐	☐
Dyslipidemia (cholesterol, triglyceride)	☐	☐
Urinary tract stone disease (nephrolithiasis, Ureteolithiasis)	☐	☐
Dementia	☐	☐
Rheumatioid arthritis	☐	☐
Other disease<br>1)<br>2)<br>3)	 ☐<br>☐<br>☐	 ☐<br>☐<br>☐

・Do you take any prescription drugs regularly? (not including over-the-counter drugs, nor dietary supplement)

☐ Yes ⇒ Please check the following checkbox ☐ of drug you take.

☐ No

☐Antihypertensive drug ☐Lipid-lowering drug ☐Antidiabetic drug

☐Hypnotic ☐Antipyretic analgesic ☐Allergic therapeutic drug ☐Antianginal drug

☐laxative ☐Antiosteoporosis drug ☐Antirheumatic drug ☐Adrenocorticosteroid

☐Antibiotic ☐Gastric ulcer therapeutic drug ☐Dementia therapeutic drug

☐Cold remedy (drug name:)

☐Chinese herbal drug (drug name:)

☐Other drug (drug name:)

Smoking history

・Please choose your smoking status from the following choices.

☐ Never smoker

☐ Currently smoker

☐ Past smoker
