# Supplementary material for: Association of single nucleotide polymorphisms in the NRF2 promoter with vascular stiffness with aging
Source: PLoS One. 2020 Aug 11;15(8):e0236834. doi: 10.1371/journal.pone.0236834 (PMC7418968; doi:10.1371/journal.pone.0236834)
Supplement: S1 Table — a P values were calculated by χ2 test. (PDF) [file pone.0236834.s002.pdf]

S1 Table. The frequency of the *NRF2* SNP (−617C>A) stratified by gender

Characteristics	Total	n (%)			*P* value[a]
		CC	CA	AA	
**All participants**					
Total	1800	927 (51.5)	724 (40.2)	149 (8.3)	
Male	698	347 (49.7)	293 (42)	58 (8.3)	0.454
Female	1102	580 (52.6)	431 (39.1)	91 (8.3)	
**Never-smoking healthy subjects**					
Total	642	340 (53)	247 (38.5)	55 (8.6)	
Male	138	68 (49.3)	60 (43.5)	10 (7.2)	0.377
Female	504	272 (54)	187 (37.1)	45 (8.9)	

[a] *P* values were calculated by X^2test
