# Supplementary material for: Association of single nucleotide polymorphisms in the NRF2 promoter with vascular stiffness with aging
Source: PLoS One. 2020 Aug 11;15(8):e0236834. doi: 10.1371/journal.pone.0236834 (PMC7418968; doi:10.1371/journal.pone.0236834)
Supplement: S2 Table — a P values were calculated by Kruskal-Wallis (K.W.) test with post-hoc test by Holm. Abbreviation: IQR, interquartile range; baPWV: bronchial-ankle pulse wave velocity, ABI: ankle branchial pressure index, SBP: systolic blood pressure, DBP: diastolic blood pressure, MAP: mean arterial pressure, PP: pulse pressure. (PDF) [file pone.0236834.s003.pdf]

S2 Table. The median and IQR of four-limb blood pressure measurements in never-smoking healthy subjects

Characteristics		median/IQR			*P* value[a]			
		CC	CA	AA	K.W.	CC vs CA	CC vs AA	CA vs AA
**Total**								
Number of subjects		340	247	55				
Age		44/34-59	46/34-62	51/34-61	0.460	-	-	-
baPWV	(cm/s)	1230/1092-1450	1285/1106-1510	1317/1169-1589	0.030	0.188	0.047	0.188
ABI ×100		111/107-116	111/106-117	112/108-116	0.837	-	-	-
SBP	(mmHg)	116/107-127	120/110-134	120/111-135	0.003	0.006	0.059	0.675
DBP	(mmHg)	69.5/61.9-76.5	71.9/63.8-79.9	70.3/63.2-84.5	0.042	0.077	0.195	0.698
MAP	(mmHg)	87.9/79.5-97.7	90.5/82.0-103	91.2/84.5-105	0.010	0.037	0.046	0.368
PP	(mmHg)	47.5/42.8-53.9	48.9/43.5-57.1	48.5/45.1-56.1	0.017	0.042	0.086	0.625
**Male**								
Number of subjects		68	60	10				
Age		40/32-56	38.5/32-57	37/30-51	0.940	-	-	-
baPWV	(cm/s)	1262/1192-1412	1319/1155-1471	1223/1208-1482	0.781	-	-	-
ABI ×100		114/108-119	113/107-120	110/109-119	0.930	-	-	-
SBP	(mmHg)	119/114-128	123/117-136	120/117-134	0.061	0.058	0.755	0.755
DBP	(mmHg)	71.5/65.1-78.6	75.1/68.3-87.7	70.0/64.6-77.9	0.080	0.073	0.911	0.793
MAP	(mmHg)	88.5/83.9-97.6	93.0/84.6-109	90.5/87.9-101	0.126	-	-	-
PP	(mmHg)	48.5/43.8-54	48.9/44.0-55.3	51.7/46.6-55.2	0.370	-	-	-
**Female**								
Number of subjects		272	187	45				
Age		46/35-60	50/35-62	54/37-62	0.260	-	-	-
baPWV	(cm/s)	1210/1075-1456	1273/1085-1514	1338/1089-1609	0.039	0.241	0.049	0.241
ABI ×100		111/107-116	111/106-114	112/108-116	0.660	-	-	-
SBP	(mmHg)	115/106-127	118/108-133	120/109-136	0.028	0.094	0.094	0.436
DBP	(mmHg)	68.5/60.6-75.9	70/61.8-78.0	71.0/62.1-85	0.151	-	-	-
MAP	(mmHg)	87.8/78.5-97.7	90/80.2-102	92.5/84.4-106	0.048	0.184	0.102	0.230
PP	(mmHg)	47.1/42.5-53.6	48.9/43.3-57.7	48/44.7-56.2	0.038	0.065	0.196	0.824

[a] *P* values were calculated by Kruskal-Wallis (K.W.) test with post-hoc test by Holm.

Abbreviation: IQR, interquartile range; baPWV: bronchial-ankle pulse wave velocity, ABI: ankle branchial pressure index, SBP: systolic blood pressure, DBP: diastolic blood pressure, MAP: mean arterial pressure, PP: pulse pressure
