# Supplementary material for: Association of single nucleotide polymorphisms in the NRF2 promoter with vascular stiffness with aging
Source: PLoS One. 2020 Aug 11;15(8):e0236834. doi: 10.1371/journal.pone.0236834 (PMC7418968; doi:10.1371/journal.pone.0236834)
Supplement: S3 Table — a P values were calculated by Kruskal-Wallis (K.W.) test with post-hoc test by Holm. Abbreviations: IQR, interquartile range; AST, aspartate transaminase; ALT, alanine aminotransferase; γ-GTP, gamma-glutamyl transpeptidase; BUN, blood urea nitrogen; HbA1c, glycated hemoglobin; HOMA-IR, homeostasis model assessment of insulin resistance; HDL, high-density lipoprotein-cholesterol; LDL, low-density lipoprotein-cholesterol; L/H ratio, LDL/HDL ratio. (PDF) [file pone.0236834.s004.pdf]

S3 Table. The median and IQR of general characteristics in female never-smoking healthy subjects

Characteristics		median/IQR CC	CA	AA	*P* value[a] K.W.	CC vs CA	CC vs AA	CA vs AA
Number of subjects		272	187	45				
Age		46/35-60	50/35-62	54/37-62	0.260	-	-	-
Erythrocyte	(10,000/μl)	436/419-456	439/418-464	433/409-451	0.335	-	-	-
Hemoglobin	(g/dL)	13/12.3-13.6	13/12.4-13.6	12.8/12.2-13.3	0.739	-	-	-
Hematocrit	(%)	41.5/39.7-43.2	41.7/39.5-43.2	41.1/39.7-42.5	0.739	-	-	-
Total protein	(g/dL)	7.3/7.0-7.5	7.3/7.0-7.5	7.3/7.0-7.7	0.461	-	-	-
AST	(IU/L)	18/16-23	19/16-23	19/17-21	0.785	-	-	-
ALT	(IU/L)	14/11-19	14/11-20	14/11-17	0.690	-	-	-
γ-GTP	(IU/L)	16/13-23	15/13-20	17/13-21	0.460	-	-	-
BUN	(mg/dL)	13.1/10.8-15.7	12.9/10.3-15.1	12.1/9.8-15.8	0.29	-	-	-
Creatinine	(mg/dL)	0.61/0.57-0.66	0.62/0.55-0.67	0.57/0.52-0.64	0.0432	0.811	0.0298	0.0694
Fasting glucose	(mg/dL)	78/72-85	78/73-84.5	79/76-85	0.528	-	-	-
HbA1c	(%)	5.6/5.4-5.8	5.6/5.4-5.8	5.5/5.3-5.7	0.265	-	-	-
Insulin	(μIU/mＬ)	4.0/3.1-5.2	4.1/3.1-5.4	4.1/3.0-5.5	0.780	-	-	-
HOMA-IR		0.75/0.58-1.03	0.82/0.55-1.13	0.80/0.56-1.11	0.862	-	-	-
C-peptide	(ng/mL)	0.9/0.7-1.0	0.9/0.7-1.1	0.8/0.7-1.1	0.690	-	-	-
Triglyceride	(mg/dL)	62/45-87	64/48-88	67/53-89	0.41	-	-	-
Total cholesterol	(mg/dL)	196/172-221	198/171-216	205/177-223	0.439	-	-	-
HDL cholesterol	(mg/dL)	70/60-80	66/56-76	69/63-85	0.0291	0.0446	0.633	0.139
LDL cholesterol	(mg/dL)	112/91-132	111/93-131	115/94-132	0.837	-	-	-
L/H ratio		1.56/1.24-1.99	1.68/1.37-2.06	1.59/1.25-1.96	0.0908	0.094	0.798	0.563
Iron	(μg/dL)	97/71-120	91/67-115	92/70-120	0.319	-	-	-
Ferritin	(ng/mL)	39.0/17.1-74.4	35.6/13.7-64.9	36.7/11.7-73	0.709	-	-	-
Total bilirubin	(mg/dL)	0.8/0.6-1.0	0.8/0.6-1.0	0.9/0.7-1.1	0.216	-	-	-

[a] *P* values were calculated by Kruskal-Wallis (K.W.) test with post-hoc test by Holm.

Abbreviations: IQR, interquartile range; AST, aspartate transaminase; ALT, alanine aminotransferase; γ-GTP, gamma-glutamyl transpeptidase; BUN, blood urea nitrogen; HbA1c, glycated hemoglobin; HOMA-IR, homeostasis model assessment of insulin resistance; HDL, high-density lipoprotein-cholesterol; LDL, low-density lipoprotein-cholesterol; L/H ratio, LDL/HDL ratio.
