# Supplementary material for: Association of single nucleotide polymorphisms in the NRF2 promoter with vascular stiffness with aging
Source: PLoS One. 2020 Aug 11;15(8):e0236834. doi: 10.1371/journal.pone.0236834 (PMC7418968; doi:10.1371/journal.pone.0236834)
Supplement: S4 Table — a P values were calculated by Kruskal-Wallis (K.W.) test with post-hoc test by Holm. Abbreviations: IQR, interquartile range; AST, aspartate transaminase; ALT, alanine aminotransferase; γ-GTP, gamma-glutamyl transpeptidase; BUN, blood urea nitrogen; HbA1c, glycated hemoglobin; HOMA-IR, homeostasis model assessment of insulin resistance; HDL, high-density lipoprotein-cholesterol; LDL, low-density lipoprotein-cholesterol; L/H ratio, LDL/HDL ratio. (PDF) [file pone.0236834.s005.pdf]

S4 Table. The median and IQR of general characteristics in male never-smoking healthy subjects

Characteristics		median/IQR			*P* value[a]			
		CC	CA	AA	K.W.	CC vs CA	CC vs AA	CA vs AA
Number of subjects		68	60	10				
Age		40/32-56	39/32-57	37/30-51	0.940	-	-	-
Erythrocyte	(10,000/μl)	492/472-511	493/466-518	483/470-540	0.919	-	-	-
Hemoglobin	(g/dL)	14.9/14.3-15.5	14.9/14.4-15.4	15.1/14.175-15.8	0.933	-	-	-
Hematocrit	(%)	46.6/45.5-48.6	46.6/44.6-48.5	45.9/43.4-48.5	0.802	-	-	-
Total protein	(g/dL)	7.4/7.2-7.6	7.4/7.1-7.6	7.5/7.3-7.7	0.492	-	-	-
AST	(IU/L)	21/19-25	22/17-25	21/17-30	0.743	-	-	-
ALT	(IU/L)	20/16-31	21/16-30	18/16-24	0.814	-	-	-
γ-GTP	(IU/L)	26/18-40	27/18-50	23/16-40	0.645	-	-	-
BUN	(mg/dL)	13.4/12.0-16.3	14.4/12.9-16.8	13.6/13.0-14.3	0.256	-	-	-
Creatinine	(mg/dL)	0.82/0.75-0.87	0.80/0.74-0.89	0.80/0.68-0.85	0.763	-	-	-
Fasting glucose	(mg/dL)	78/72-85	79/74-88	82/74-87	0.746	-	-	-
HbA1c	(%)	5.5/5.3-5.7	5.5/5.4-5.7	5.8/5.5-5.9	0.257	-	-	-
Insulin	(μIU/mＬ)	4.4/2.9-5.2	4.1/2.8-5.8	3.8/2.6-4.7	0.871	-	-	-
HOMA-IR		0.85/0.51-1.08	0.83/0.54-1.12	0.75/0.51-0.96	0.886	-	-	-
C-peptide	(ng/mL)	0.9/0.8-1.1	1.0/0.8-1.3	0.8/0.7-0.9	0.236	-	-	-
Triglyceride	(mg/dL)	94/56-132	85/56-133	69/60-111	0.717	-	-	-
Total cholesterol	(mg/dL)	186/169-217	191/170-212	202/161-221	0.914	-	-	-
HDL cholesterol	(mg/dL)	58/48-65.3	58.5/48-67	62/51-79	0.509	-	-	-
LDL cholesterol	(mg/dL)	106/86-127	105/88-125	95/87-115	0.761	-	-	-
L/H ratio		1.83/1.43-2.36	1.77/1.40-2.52	1.63/1.06-1.91	0.595	-	-	-
Iron	(μg/dL)	100/84-117	108/84-136	111/100-131	0.181	-	-	-
Ferritin	(ng/mL)	132.5/97.8-176.3	154.5/91.5-216.5	100.6/74.5-123.3	0.090	0.222	0.346	0.222
Total bilirubin	(mg/dL)	0.8/0.7-1.0	0.9/0.7-1.1	0.8/0.7-0.9	0.635	-	-	-

[a] *P* values were calculated by Kruskal-Wallis (K.W.) test with post-hoc test by Holm.

Abbreviations: IQR, interquartile range; AST, aspartate transaminase; ALT, alanine aminotransferase; γ-GTP, gamma-glutamyl transpeptidase; BUN, blood urea nitrogen; HbA1c, glycated hemoglobin; HOMA-IR, homeostasis model assessment of insulin resistance; HDL, high-density lipoprotein-cholesterol; LDL, low-density lipoprotein-cholesterol; L/H ratio, LDL/HDL ratio.
