# Supplementary material for: Association of single nucleotide polymorphisms in the NRF2 promoter with vascular stiffness with aging
Source: PLoS One. 2020 Aug 11;15(8):e0236834. doi: 10.1371/journal.pone.0236834 (PMC7418968; doi:10.1371/journal.pone.0236834)
Supplement: Attachment — Submitted filename: Shimizu_et_al._Response to Reviewers.docx [file pone.0236834.s006.docx]

**Re**: PONE-D-20-12583

Dr. Hiroyuki Itabe

Academic Editor

PLOS ONE

Dear Dr. Itabe,

Thank you for your email of 27th May 2020 regarding our manuscript entitled "Association of single nucleotide polymorphisms in the NRF2 promoter with vascular stiffness with aging " and for providing us an opportunity to revise it.

We appreciate for the insightful and straightforward comments from the reviewers. Responding to the reviewers’ comments, we added the description to fully discuss the reviewers’ concerns in the “Discussion” section. As a result, we believed that the manuscript has significantly improved. Please refer to the following “response to the reviewers’ comments” for the details.

I appreciate for your kind consideration of the revised manuscript and look forward to hearing from you at your earliest convenience.

Yours sincerely,

Ken Itoh, MD & PhD

Hirosaki University Graduate School of Medicine

5 Zaifu-cho, Hirosaki 036-8562, Japan

Tel.: 81-172-39-5158

Fax: 81-172-39-5157

E-mail: [itohk@hirosaki-u.ac.jp](mailto:itohk@hirosaki-u.ac.jp)

**Response to reviewers’ comments**

**Reviewer #1**

Authors conducted well designed study with enough subject number. Results from their study enhanced new insight into roll of Nrf2 in atherosclerosis. This reaches standards of published in peer-reviewed journal. Thank you.

**Response:** Thank you for the comment and we appreciate for your evaluation.

**Reviewer #2**

The authors examined baPWV, an indicator of vascular stiffness and SNPs in the NRF gene SNP-617 and showed the clear association of NRF activity and arterial stiffness. However, some issues written below are still concerned.

**Response:** Thank for your evaluation of the manuscript and insightful and straightforward comments.

Major:

1. The authors showed the clear relationship between baPWA and SNP-617 in people under 60 years old. However, if it is true, this relationship should become clearer in people over 60 years old, which may become more important in terms of prevention of cardiovascular disease.

**Response:** Thank you for the comment. We consider that there are two main reasons why the relationship between baPWV, SNP−617 and age are not significant over 60 years old. One is selection bias and the other is insufficient statistical power. Accordingly, we added the description in the Discussion section (page 25, sentence 430 to 440 in the revised manuscript with track change).

2. Although the authors described in the limitation of the study, it is not clear whether there is a gender difference in the results of baPWV, SBP, MAP and PP.

**Response:** Thank you for the comment. Because the results in male contain similar analytical limitations as the analysis of the older subjects (i. e. selection bias and insufficient statistical power), we consider that this study couldn’t reveal gender differences in relationship between SNP−617 and baPWV. Therefore, we added the explanation in Discussion section (page 24, sentence 407 to 420 in the revised manuscript with track change)

3. It is relatively easy to understand there is a relationship between SNP-617 and arterial stiffness, but hard to connect it to creatinine and HDL cholesterol levels.

**Response:** Thank you for the comment. We have added some additional discussion about the results of creatinine and HDL cholesterol in the Discussion section (page 23, sentence 383 to 403 in the revised manuscript with track change).

Minor:

1. P6, l11 oxidative stress: under the condition of oxidative stress?

**Response:** Thank you for the comment. We have corrected the manuscript.

2. P10, l5 SBP pressure: delete pressure

**Response:** Thank you for the comment. We have corrected the manuscript.

3. P13, l14 CA, 1317/1169-1589: change to AA,

**Response:** Thank you for the comment. We have corrected the manuscript.

4. P14, l16 *p<0.01: change to **p<0.01

**Response:** Thank you for the comment. We have corrected the manuscript.

5. P22, l16 creatinine HDL cholesterol: insert and between creatinine and HDL cholesterol

**Response:** Thank you for the comment. We have corrected the manuscript.

6. P22, l18 AA, CA, and CC: change to AA, CA and CC

**Response:** Thank you for the comment. We have corrected the manuscript.
